# Double-Blind, Randomized, Three-Armed, Placebo-Controlled, Clinical Investigation to Evaluate the Benefit and Tolerability of Two Dosages of IQP-AE-103 in Reducing Body Weight in Overweight and Moderately Obese Subjects

**DOI:** 10.1155/2019/3412952

**Published:** 2019-02-03

**Authors:** Ralf Uebelhack, Udo Bongartz, Stephanie Seibt, Gordana Bothe, Pee Win Chong, Patricia De Costa, Natalia Wszelaki

**Affiliations:** ^1^analyze & realize GmbH, Weißenseer Weg 111, 10369 Berlin, Germany; ^2^analyze & realize GmbH, Waldseeweg 6, 13467 Berlin, Germany; ^3^Zaluvida Corporate Sdn Bhd, E-16 Plaza Mont Kiara, 2 Jalan Kiara, 50480 Kuala Lumpur, Malaysia; ^4^InQpharm Group Sdn Bhd, E-16 Plaza Mont Kiara, 2 Jalan Kiara, 50480 Kuala Lumpur, Malaysia

## Abstract

**Objective:**

This study was performed to determine the efficacy and tolerability/safety of IQP-AE-103 on body weight reduction in overweight to moderately obese adults.

**Methods:**

A double-blind, randomized, placebo-controlled trial involved one hundred and eight subjects (BMI between 25 and 35 kg/m^2^) that were randomly assigned to either the low-dose or the high-dose IQP-AE-103 group, or the placebo group. Following a 2-week run-in period, subjects received two capsules of investigational product after three daily main meals for 12 weeks. Subjects were instructed to maintain a nutritionally balanced hypocaloric diet according to the individual's energy requirement. Body weight, body fat, and waist and hip circumference were measured at baseline, and after 2, 4, 8, and 12 weeks. Subjects also rated their feelings of hunger and fullness using visual analogue scales, and food craving on a 5-point scale at the same time intervals. Blood samplings for safety laboratory parameters were taken before and at the end of the study.

**Results:**

After 12 weeks of intake, the high-dose IQP-AE-103 group had a significantly greater weight loss compared with the placebo (5.03 ± 2.50 kg vs. 0.98 ± 2.06 kg, respectively; *p* < 0.001) and the low-dose group (3.01 ± 2.19 kg; *p*=0.001). The high-dose group experienced a decrease in body fat of 3.15 ± 2.41 kg compared with a decrease of 0.23 ± 2.74 kg for the placebo group (*p* < 0.001). High-dose IQP-AE-103 also decreased the feeling of hunger in 66% subjects. A beneficial effect of IQP-AE-103 on the lipid metabolism was also demonstrated in the subgroup of subjects with baseline total cholesterol levels above 6.2 mmol/L. No side effects related to the intake of IQP-AE-103 were reported.

**Conclusions:**

These findings indicate that IQP-AE-103 could be an effective and safe weight loss intervention. This trial is registered with NCT03058367.

## 1. Introduction

The growing prevalence of overweight and obesity is a persistent international problem. Overweight (BMI from 25.0 to 29.9) and obesity (BMI of 30 and higher) are defined as excessive accumulation and storage of fat in the body. Obesity is a serious chronic disease that has far-ranging negative implications on many systems in the human body. With the rise of BMI and body weight, the risks for type 2 diabetes, fatty liver, ischemic heart disease, hypertension, stroke, obstructive sleep apnea, and certain cancers such as breast and colon cancers grow significantly [1–4]. Obesity was also found to increase the risk of depression caused by disparagement of body image and negative emotional reaction to dieting [5], leading to job absenteeism, lowering productivity, and driving healthcare costs [6, 7]. In the United States alone, obesity-related diseases medical care costs up to $210 billion annually [8]. Therefore, successful and sustainable body weight management is not only crucial for human health but also for the economy. As per recently published public health guidelines of National Institute for Health and Care Excellence and a report of the American College of Cardiology/American Heart Association Task Force on Practice Guidelines and the Obesity Society, losing 3% of baseline body weight is already associated with health benefits [9–11]. Moderate weight loss of 5% was shown to decrease the plasma concentrations of some risk factors for cardiometabolic disease and obstructive sleep apnea (glucose, insulin, triglyceride, alanine transaminase, HDL cholesterol, and leptin) [10–13].

There are many factors contributing to overweight or obesity such as physical inactivity, poor diet, high calorie intake, genetics, or the way the body uses energy. Hence, reducing body weight and calorie intake, introducing balanced diet, and increasing physical activity are critical to achieve weight loss. Pharmaceutical drug therapies are indicated when lifestyle intervention alone is unsuccessful. However, the vast majority of the currently available therapies are associated with unpleasant or even serious adverse effects. Many anti-obesity drugs that have been approved by health authorities have eventually been withdrawn from the market due to serious adverse events, for example, sibutramine or rimonabant, leaving limited options, for example, orlistat. But orlistat has also a long list of side effects, such as fecal incontinence, oily spotting, diarrhea, or even more severe side effects such as serious liver injury [14–16]. Since weight loss is a long-term treatment, anti-obesity medications should have good tolerability and be free of side effects. For this reason, new strategies have to be developed to combat this complex health condition.

Plants have always been a good source of new potential and natural therapies for illnesses, including body weight management products. Litramine®, a natural fibre complex derived from *Opuntia ficus-indica*, enriched with soluble fibres from *Acacia* sp., has been shown in a number of clinical trials to safely and effectively reduce and maintain weight loss [17–20]. Litramine® has been demonstrated to reduce dietary fat absorption and increase fecal fat excretion, resulting in lower calorie absorption from diet, which leads to weight loss [20, 21].

IQP-AE-103 is a combination of dehydrated powder of okra (*Abelmoschus esculentus* (L.) Moench) pods and inulin, a heterogeneous mixture of fructose polymers extracted from chicory roots. Okra pods are a widely consumed vegetable, eaten raw or cooked, used in salads, soups, and stews. In stews, okra acts also as a thickening agent, increasing the viscosity of the liquid. Research by Bakre and Jaiyeaoba demonstrated that dehydrated powder derived from okra pods has high swelling capabilities [22]. Due to this physicochemical property, okra pods can potentially induce satiety and fullness and help to control the calorie intake [22, 23]. Additionally, okra pods contain dietary fibre and proteins [23], which may play a role in fat binding, thus decreasing absorption of dietary fat and consequently leading to body weight reduction [24]. Inulin is a generic name covering all *β*-(2 → 1) fructans. The presence of these bonds makes inulin indigestible by the human enzymes. Inulin is, therefore, associated with low caloric value. However, these bonds are fermentable by colonic bacteria; thus, inulin can be considered a good source of prebiotics [25, 26]. The health benefits of inulin have been demonstrated in a number of clinical studies and include weight loss [27], modulation of intestinal/fecal microflora [28, 29], and improvement of bowel movements in elderly constipated subjects [29].

In addition, a combination of dehydrated okra pod powder and inulin, the active ingredients in IQP-AE-103, has been shown to be a strong fat-binding agent in an *in vitro* setting simulating *in vivo* conditions (data not shown); it also exerts high swelling capacity and significantly enhances solution viscosity. Thus, IQP-AE-103 appears to be a promising natural agent that may help to control calorie intake, decrease absorption of dietary fat, and consequently lead to body weight reduction.

In this study, we investigated the efficacy and tolerability/safety of IQP-AE-103 for reducing body weight in overweight and obese subjects.

## 2. Materials and Methods

### 2.1. Participants

One hundred and eight (108) generally healthy, overweight, to moderately obese (BMI ≥25 and <35 kg/m^2^) male and female volunteers between 18 and 65 years of age participated in the study. Other inclusion criteria were a consistent and stable body weight for 3 months prior to recruitment, being accustomed to 3 meals daily, commitment to adhere to the study's dietary recommendation, use of contraception in women of childbearing potential, maintenance of habitual level of physical activity during the study, and avoiding other weight management products or programs. Main exclusion criteria included hypersensitivity to the ingredients, pregnancy or nursing, smoking cessation within 6 months prior to the study, abuse of drugs and alcohol, diabetes mellitus type 1, uncontrolled diabetes mellitus type 2, relevant endocrine disorders (e.g., uncontrolled thyroid disorders), eating disorders, recent history of gastrointestinal surgery, acute and chronic gastrointestinal disorders, and use of medications that may affect body weight or gastrointestinal functions. All subjects gave written informed consent voluntarily. The clinical trial was approved by the ethics committee of Charité–University Medicine Berlin and was performed in compliance with principles of the World Health Organization (Declaration of Helsinki) [30] and the EU recommendations for Good Clinical Practice (EMA/CHMP/ICH/135/95), E6 (R2) [31].

### 2.2. Interventions

Subjects underwent a 2-week run-in phase to assess the compliance to the study requirements and the recommended dietary regimen, before randomization for the 12-week treatment phase. Subjects were randomly allocated to either IQP-AE-103 high dose, IQP-AE-103 low dose, or the placebo group according to the randomization code provided by an independent statistician. Subjects were instructed to take two capsules of the investigational product three times daily, 15 minutes after each main meal (breakfast, lunch, and dinner), with a glass of water (250 ml). Each capsule of IQP-AE-103 high dose contained 330 mg dehydrated okra powder and 85 mg inulin, whereas low-dose capsules contained half the amount of okra powder and inulin; and placebo capsules contained standard excipients. All the capsules were identical in size and appearance. Dehydrated okra powder was obtained from whole okra pods (*Abelmoschus esculentus*) that were cleaned, sliced, oven-dried at 60°C, and milled to form a fine powder. Inulin was extracted from freshly sliced chicory roots using hot water, followed by filtration and purification by discoloration and decalcification. The materials were tested for microbiological activity and contaminants, and inulin was tested for purity. The daily dosage of IQP-AE-103 was determined based on data obtained from a previous animal study that has shown efficacy in preventing weight gain and in increasing fecal excretion (data not shown). Considering the mode of action of the product, that is, reducing dietary fat absorption, the daily dosage was divided into 3 equal doses to be consumed after 3 main meals.

Throughout the study, all subjects were instructed to follow a nutritionally balanced and hypocaloric diet plan, containing 30% fat, developed by a registered dietician. The daily basal energy requirement was estimated for each subject according to the Institute of Medicines' equations taking into considerations sex, age, and actual body weight, and multiplying by a factor for physical activity level [32]. The estimated energy requirement was then reduced by 20% in order to achieve a mildly hypocaloric diet. Each subject was issued a diet plan according to individual energy requirement and given instruction to follow the diet plan. A sample of diet plan is shown in Table 1. Subjects had to record the items of the diet plan consumed, along with their calorie content, in a diary on 2 weekdays and 1 day of the weekend per week.

### 2.3. Measurements

Subjects were followed up through scheduled visits with investigators after 2, 4, 8, and 12 weeks of interventions. Given the short intervals between the visits, no regular phone calls were scheduled; however, the subjects were free to contact the study sites as needed. Body weight was measured in barefoot subjects wearing underwear at each visit, whereas body composition was measured by bioimpedance method; both measurements were taken using validated electronic weighing scale (Tanita BC-420 MA). Subjects' standing height was measured with a wall-mounted stadiometer. Waist circumference (cm) was measured at the level midway between the lateral lower rib margin and the iliac crest, whereas hip circumference (cm) was measured as the maximal circumference over the buttocks.

To provide initial insight into potential effects of the investigational product on appetite/satiety, subjects were asked to provide a retrospective subjective assessment of the overall feeling of hunger, fullness, and food craving for the last 3 days prior to the respective study visit. For the evaluation of hunger and fullness, a visual analogue scale (VAS) was used, respectively. VASs used (100 mm in length, with expressions for most negative and most positive ratings anchored at each end) were based on the original tools used in appetite research to measure the acute sensations following a test meal [33]. The retrospective assessment per se has not been validated but was used in previous research [34]. To assess the feeling of food craving, a 5-point rating scale (0 = “no,” 1 = “slightly,” 2 = “moderate,” 3 = “strong,” and 4 = “very strong”) was used to reply to a general single question if the subject had experienced any craving. Generally, a 5-point scale has been considered reliable [35]; however, the specific use for food craving rating has not been clinically validated.

Stool frequency was assessed based on bowel movements recorded in the subject diaries 3 days a week. Physical activity was recorded using validated International Physical Activity Questionnaire, Short Form (IPAQ-SF) [36] during all study visits from visit 2 to visit 6.

Blood lipid parameters, fasted glucose, and hemoglobin A1c (HbA1c) were assessed at visit 1 and visit 6.

Safety evaluation included measurement of laboratory parameters (full blood count, clinical chemistry, liver and renal functions and fat-soluble vitamins (A, D, E, and K) levels) and urine analysis at visit 1 and visit 6, as well as assessment of vital signs during each visit. Adverse events were recorded, regardless of causality at every visit. Global evaluation of tolerability by subjects and investigators was performed at the end of the study.

Compliance regarding the intake of the investigational products was evaluated by the collection of unused capsules. The overall compliance (%) was defined as (the total number of capsules taken divided by the total number of capsules to be taken) multiplied by 100. The total number of capsules to be taken was calculated per subject, taking into account the actual length of the IP consumption period.

### 2.4. Statistical Methods and Sample Size Determination

The primary endpoint of this study was the comparison of the mean body weight (kg) change between IQP-AE-103 high dose and placebo after 12 weeks of intervention from baseline, in overweight and moderately obese subjects. The evaluation of the efficacy of IQP-AE-103 was based on the null hypothesis that there are no statistical differences between IQP-AE-103 and placebo in mean reduction of body weight after 12 weeks of treatment. The nonparametric Mann–Whitney *U* test for independent samples was applied. The testing was carried out by the determination of the rank sum of individual body weight changes.

All other efficacy as well as the safety and concurrent variables were exploratively examined and were descriptively assessed. For the metric data (continuous data), the statistical characteristics were given (number, mean, standard deviation, median, extremes, quartiles, and 95% confidence interval of mean). For ordinal data (discrete data), frequency distribution was performed. All nominal data (categorical data) were summarized using frequency tables. All variables were evaluated primarily by exact nonparametric procedures:

- Kruskal–Wallis test for more than two independent groups (comparison of groups or subgroups)
- Mann–Whitney *U* test for independent groups (comparison of groups or subgroups)
- Wilcoxon test for dependent groups (comparison of pre-post within groups or subgroups)
- Fisher's exact test for comparison of percentage.

For all statistical analyses, the level of significance (*p* < 0.05) was assumed. Efficacy and safety data were analyzed based on full analysis set (FAS) population, which was defined as subjects who have received at least one dose of the investigational product and had a baseline and at least one postbaseline assessment. All statistical analyses were done using the SPSS statistic software, V22.0 (SPSS, Chicago, IL).

The sample size estimation was determined by the Cohen's effect size (group comparison), as well as the previously determined requirements of the significance level of 5.0% (two tailed) and power of 80%. As no clinical data were available for IQP-AE-103 at the study initiation that may be used as basis for the sample size calculation, estimation was based on a comparable clinical trial [37]. In this study, the body weight reduction after 12 weeks of the treatment with verum represented 3.8 ± 1.8 kg compared with placebo (1.4 ± 2.6 kg). The difference of 2.4 kg reflects a large effect (effect size of 1.0). This effect size was considered suitable for a sample size calculation of the present study. In consideration of the nonparametric test procedure, the necessary sample size was equal to 28 subjects per study arm. Considering the dropout rate of 20% and the block randomization with the block size of 6, a total sample size of 36 subjects per study group (total 108 subjects) was recommended. No dropout was replaced.

## 3. Results

**Figure 1:**
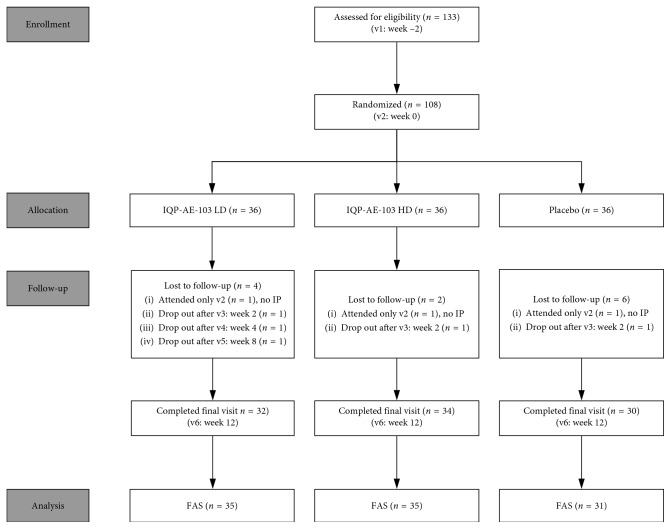
Flowchart of the study population. IP = investigational product, LD = low dose, HD = high dose, v = visit, FAS = full analysis set.

This double-blind, randomized, placebo-controlled study was conducted between April and November 2017. One hundred and eight (108) subjects were randomized into three groups. Seven subjects were excluded from the full analysis set (FAS) and the safety population as they attended only baseline visit and did not receive any investigational product. Flowchart of the study population is given in Figure 1. The baseline characteristics of the three study groups are given in Table 2. There was no statistically significant difference between the study groups in gender distribution, age, body height, body weight, BMI, energy requirement, waist circumference, hip circumference, body fat mass, and fat-free mass. Mean subject age was 47.5 ± 12.4 years, with a higher percentage of female subjects than male subjects in each treatment group. 107 subjects were Caucasian, whereas for one subject, the ethnicity was neither Caucasian nor Asian, however not further specified. The compliance with the intake of the investigational product was assessed on subjects who completed all study visits. 99.1% (*n*=31) subjects were compliant in the low-dose group and 99.0% (*n*=34) in the high-dose group vs. 99.9% (*n*=30) subjects in the placebo group. There were no statistically significant differences in the intake of the investigational product related to the capsules consumed between the study groups (*p*=0.922).

**Figure 2:**
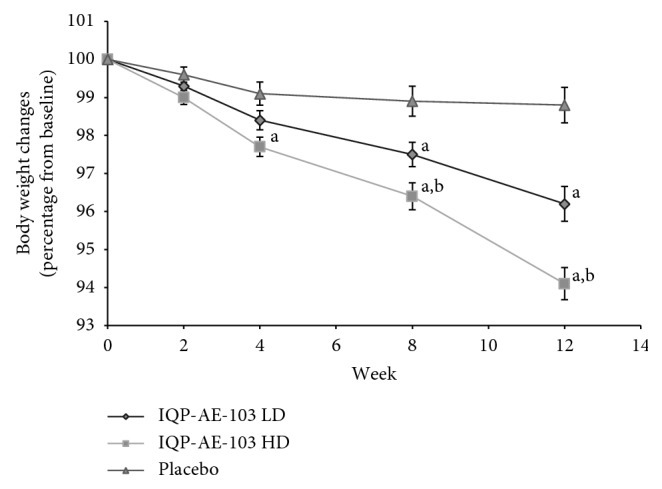
Mean body weight over time in percentage relative to baseline weight. LD = low dose; HD = high dose. Error bars denote standard error of mean. (a) significant vs. placebo and (b) significant vs. IQP-AE-103 LD.

**Figure 3:**
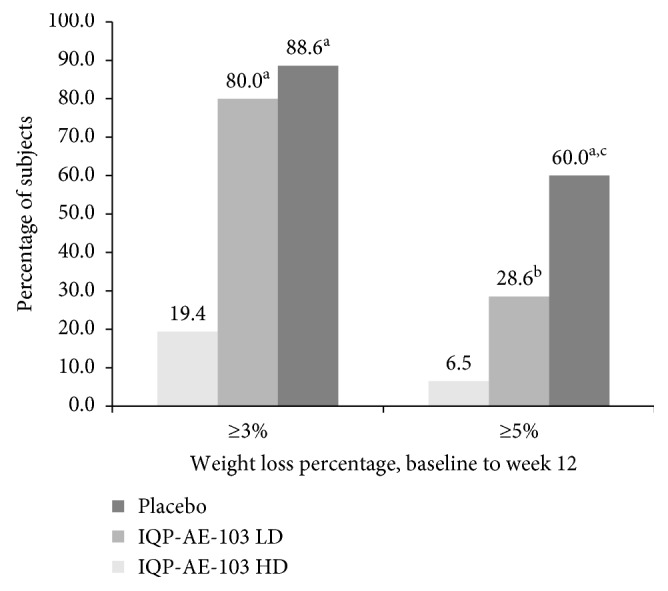
Responder rate for subjects who lost ≥3% and ≥5% of initial body weight at v6. LD = low dose; HD = high dose. ^a^*p* < 0.001 vs. placebo; ^b^*p*=0.026 vs. placebo; ^c^*p*=0.015 vs. low-dose group.

At baseline, the study groups did not differ in the body weight. However, body weight significantly decreased from baseline to week 12 in the high-dose IQP-AE-103 vs. placebo group, 5.03 ± 2.50 kg vs. 0.98 ± 2.06 kg (*p* < 0.001). A significant reduction in body weight was also observed in the low-dose IQP-AE-103 group (3.01 ± 2.19 kg) compared to placebo (*p* < 0.001). The difference in body weight reduction between the high- and the low-dose groups of IQP-AE-103 is also significant (*p*=0.001). Mean body weight changes over the study course are presented in Figure 2 (percentage relative to baseline) and Table 3 (kg loss from baseline), respectively. Mean changes in primary and secondary parameters between baseline and week 12 are summarized in Table 4. Body weight reduction for high-dose IQP-AE-103 was observed as early as at week 2 (0.87 ± 1.16 vs. 0.34 ± 0.95, *p*=0.069 for comparison with placebo, strong trend) and achieved significance versus placebo from week 4 (1.91 ± 1.39 kg vs. 0.78 ± 1.44 kg, *p*=0.004) and onwards. In addition, at week 12, a total of 88.6% subjects in the high-dose IQP-AE-103 group lost at least 3% body weight in contrast to only 19.4% in the placebo arm (*p* < 0.001), whereas in the low-dose IQP-AE-103 group, the responder rate was 80% (*p* < 0.001 vs. placebo). Moreover, at least 5% weight loss was observed for 60% of subjects in the high-dose IQP-AE-103 group, in comparison to 28.6% in the low-dose group (*p*=0.015) and 6.5% in the placebo group (*p* < 0.001). Responder rates are presented in Figure 3. The subgroup of overweight subjects (BMI 25 to <30 kg/m^2^) had a significant weight loss in both low-dose (3.33 ± 1.70 kg, *p* < 0.001) and high-dose IQP-AE-103 groups (4.72 ± 2.33 kg, *p* < 0.001), compared with the placebo group (0.95 ± 2.03 kg), each after 12 weeks of treatment. In moderately obese subjects (BMI 30 to <35 kg/m^2^), body weight was significantly reduced in comparison with placebo only in the high-dose IQP-AE-103 group (5.39 ± 2.72 kg vs. 1.01 ± 2.16 kg; *p* < 0.001) after 12 weeks. Relative to the baseline body weight, overweight subjects in the high-dose group lost on average 5.8% of their body weight at the end of the study, whereas the low-dose group and the placebo group lost 4.3% and 1.3%, respectively. Moderately obese subjects on the other hand lost on average 6.1%, 2.5%, and 1.1% for high-dose, low-dose, and placebo groups, respectively. Subgroup analysis of body weight loss according to age groups was also performed. For subjects aged 18–40 years, high-dose IQP-AE-103 resulted in 3.71 ± 2.30 kg weight loss, compared with 1.40 ± 2.94 kg in low-dose group and 0.82 ± 2.16 kg in placebo group, after 12 weeks. Statistically significant difference in weight loss was only showed comparing high-dose and placebo groups, *p*=0.003. On the other hand, for subjects aged 41–65 years, high-dose IQP-AE-103 resulted in 5.48 ± 2.44 kg weight loss, compared with 3.56 ± 1.57 kg in low-dose group and 1.06 ± 2.06 kg in placebo group, over the same duration. Both high- and lose-dose IQP-AE-103 groups showed a significant weight loss when compared with the placebo group (*p* < 0.001 for both comparisons).

The mean reduction in waist circumference was more pronounced in both IQP-AE-103 groups, compared with that in the placebo group at the end of the study. Subjects in the high-dose IQP-AE-103 arm had a mean reduction in waist circumference of 4.1 ± 3.3 cm, whereas the mean reduction in the low-dose IQP-AE-103 arm was 2.5 ± 2.4 cm, in contrast to only 0.9 ± 1.6 cm reported in the placebo group (*p* < 0.001 and *p*=0.001 versus placebo, respectively). Also, there were statistically significant differences in the percentage of subjects with reduction in hip circumference between the study groups at week 12, between high-dose IQP-AE-103 and the placebo group (91.4% vs. 54.8%; *p*=0.001), as well as between low-dose IQP-AE-103 and placebo groups (80% vs. 54.8%; *p*=0.036), however, not between low- and high-dose IQP-AE-103 groups (*p*=0.306). No statistically significant differences in waist-to-hip ratio between the study groups were reported at week 12.

At the end of the study, significant reductions in mean BMI were seen in both high-dose (1.75 ± 0.83 kg/m^2^, *p* < 0.001) and low-dose (1.09 ± 0.75 kg/m^2^, *p* < 0.001) IQP-AE-103 subjects in comparison to the placebo group (0.34 ± 0.77 kg/m^2^).

There were statistically significant differences in reduction of body fat mass between the study groups at the end of the study (IQP-AE-103 high dose, *p* < 0.001 vs. placebo; low dose, *p*=0.005 vs. placebo and high dose vs. low dose, *p*=0.111). Reduction of fat-free mass was observed over time in all the three study groups; however, there were no significant differences between the study groups.

66% of the subjects experienced a decreased feeling of hunger in the high-dose IQP-AE-103 group. Mean VAS for feeling of hunger decreased significantly from baseline to the end of the study by 9.3 ± 25.8 mm (*p*=0.033 for pre-post change); however, the difference was not significant when compared with reduction in the placebo group (3.7 ± 22.6 mm, *p*=0.373 for pre-post change). Ratings for feeling of fullness and food cravings were not significantly changed during the course of the study in any of the study groups.

During the intervention period, no significant changes in fasting blood glucose and HbA1c levels were observed between the study groups. 67% of subjects in the IQP-AE-103 high-dose group had a reduction in triglyceride levels compared with 37% in the placebo group (*p*=0.024). Changes in total/low-density lipoprotein (LDL)/high-density lipoprotein (HDL) cholesterol were not significant at the end of the study, but a post hoc subgroup analysis performed with subjects with baseline total cholesterol level above 6.2 mmol/L revealed pre-post reductions in total cholesterol of 1.22 ± 1.10 mmol/L (*p*=0.004), 0.58 ± 0.90 mmol/L (*p*=0.047), 0.13 ± 0.31 mmol/L (*p*=0.450), and reductions in LDL cholesterol of 1.18 ± 1.06 mmol/L (*p*=0.004), 0.49 ± 0.74 mmol/L (*p*=0.044), and 0.23 ± 0.29 mmol/L (*p*=0.203), in high-dose, low-dose IQP-AE-103, and placebo groups, respectively.

Furthermore, no change in physical activity was noted by analyzing data from IPAQ-SF. Mean stool frequency did not change significantly in any of the study arms throughout the study.

At the end of the study, 97.1% of the subjects in the high-dose IQP-AE-103 group and 85.3% in the low-dose group rated the benefits of the treatment as “good” or “very good” compared with 10% in the placebo group. However, investigator rated the benefit as “good” or “very good” for 94.1% and 85.3% of the subjects in high- and low-dose IQP-AE-103 groups, respectively, compared with 6.7% for placebo subjects.

No clinically significant changes in vital signs, safety laboratory parameters (Table 5), and urine analysis were observed in the study in either of the verum groups or the placebo group at week 12. A total of 12 adverse events were reported during the course of the study (6 in high-dose IQP-AE-103 group, 4 in low-dose IQP-AE-103 group, and 2 in the placebo group), which included upper respiratory tract infections, symptoms of common cold, urinary tract infection, dental root inflammation, or lumbago. None of the reported adverse events were related to the administration of IQP-AE-103. Furthermore, tolerability was rated as “good” or “very good” by 100% of subjects in the two verum groups, comparable to 96.7% by the placebo group.

## 4. Discussion

Based on the physicochemical properties of okra pods and inulin, and the fat binding capacity shown in vitro, it was hypothesized that a product containing powdered okra pods and inulin could potentially contribute to weight loss. Hence, the primary objective of this randomised, double-blind, placebo-controlled clinical study was to evaluate the weight loss potential of IQP-AE-103 over the period of 12 weeks in overweight and moderately obese subjects. In this study, it was demonstrated that in conjunction with a hypocaloric diet, IQP-AE-103 at both low dose (990 mg okra and 255 mg inulin/day) and high dose (1980 mg okra and 510 mg inulin/day) causes significant weight loss and that the effects are greater than those in the placebo. Baseline body weight was reduced by 5.03 ± 2.50 kg in the high-dose group and by 3.01 ± 2.19 kg in the low-dose group, compared with 0.98 ± 2.06 kg in the placebo group. Also, for the high-dose group, weight loss effect due to IQP-AE-103 consumption was observed as early as at 2 weeks of treatment and was sustained over the course of the 12-week intake period. More importantly, the proportion of subjects who lost at least 5% of baseline body weight was 60.0% in the high-dose IQP-AE-103 group, a proportion that is significantly higher than that in the low-dose and the placebo groups. Such weight loss due to the intake of IQP-AE-103 is considered to be of clinical relevance as European Medicines Agency associates a weight loss of 5% or more with a decrease of disease risk factors associated with overweight and obesity [38]. Subgroup analysis showed that although both IQP-AE-103 high- and low-dose consumptions led to significant weight loss in overweight subjects, for moderately obese subjects, only high dose caused a significant body weight reduction. Subgroup analysis also showed a statistically significant weight loss in both high- and low-dose IQP-AE-103 groups in subjects aged 41–65 years when compared with placebo group, whereas only the high-dose group showed a significant weight loss versus placebo in subjects aged 18–40 years.

Direct comparisons of IQP-AE-103 with other weight loss agents that affect dietary fat absorption such as chitosan, Litramine®, or orlistat are difficult for methodological reasons. Ho et al. reported that there were no significant changes from baseline in body weight after 12 weeks of treatment with chitosan [39]. A treatment with Litramine® (a proprietary fibre complex) led to 3.8 kg weight loss after 12 weeks of intake [17]. As for orlistat (gastric and pancreatic lipase inhibitor), a study by Anderson et al. reported a 3.05 kg weight loss in overweight subjects after 16 weeks of treatment at 180 mg per day [40], whereas at higher dose of 360 mg per day, it caused a body weight reduction of 8.3 kg after 12-week period in obese women [41].

In our study, BMI, body fat mass, and waist and hip circumference were also found to be decreased significantly in both IQP-AE-103 dose groups. Lowering waist circumference, a marker of abdominal fat content, indicates that the treatment with IQP-AE-103 could potentially lower the risk of diabetes, coronary heart disease, and nonalcoholic fatty liver disease [42–44]. Additionally, the changes in fat-free mass were not significantly different between the treatment groups, indicating that the weight loss effect was primarily due to body fat reduction.

No significant differences in changes of feeling of fullness and food cravings between the study arms were observed. However, feeling of hunger decreased in 66% of subjects in the high-dose group (statistically significant pre-post change). This may be related to the swelling property of okra pods reported earlier [22]. Okra constituents such as soluble fibre or polysaccharides may increase viscosity of taken meal and form a gel, thus increasing gastric distension and thereby promoting satiety [45].

Another finding of this study is that besides weight loss, IQP-AE-103 showed beneficial effects on lipid metabolism. A significantly higher proportion of subjects in the high-dose IQP-AE-103 group experienced a reduction in triglyceride levels at the end of the study compared with placebo. In subjects with higher baseline total cholesterol levels (>6.2 mmol/L), high dose of IQP-AE-103 was shown to significantly reduce total cholesterol and LDL-cholesterol (pre-post change). Therefore, current outcome suggests that long-term treatment with IQP-AE-103 could be beneficial for subjects with elevated blood lipid levels in reducing the risk of cardiovascular disease.

Our findings stand in line with results obtained by Wang et al., who demonstrated that okra intake decreased serum and hepatic total cholesterol and triglyceride levels, and enhanced fecal excretion of bile acids in mice [46]. In other study, Kahlon et al. showed that okra polysaccharides have strong bile acid binding properties [47]. Furthermore, Chen et al. reported that okra powder showed oil-binding capacities and cholesterol adsorption properties [48]. Additionally, results from animal study by Han et al. imply that inulin may also possess a fat-binding property besides its known beneficial effect as prebiotic, which contributed to a lower triglyceride and cholesterol levels in rats fed with high fat diet in the experiments [49]. Thus, the cholesterol-lowering effect of IQP-AE-103 could possibly be related to the physicochemical properties of both okra and inulin.

IQP-AE-103 demonstrated a very good tolerability profile in our study as expected since okra is typically consumed as food and inulin is widely consumed as fibre/prebiotic supplements. Additionally, okra has shown no toxicity in animals at doses up to 4000 mg/kg [50]. A safety review on inulin by Carabin and Flamm [51] has found no treatment-related toxic effects nor carcinogenicity or genotoxicity. In our study, the overall tolerability towards the IQP-AE-103 in high and low doses was comparable to that in the placebo, according to the rating by the subjects and the investigator. Observed adverse events were unrelated with the IQP-AE-103 consumption. Moreover, there were no significant differences in stool frequency. Therefore, in contrast to many available anti-obesity products that cause unpleasant and sometimes serious side effects, IQP-AE-103 could be considered as a safe, well-tolerated, and effective weight management agent.

Our study has several limitations. One limitation is that it was performed over a short period of only 12 weeks, with no follow-up conducted after the study was finished and no weight maintenance phase after the initial weight loss. As such, the study provides no information about the effects of IQP-AE-103 on long-term weight maintenance and on other obesity-related conditions such as diabetes or cardiovascular disease. In addition, the study included subjects of a wide age range. Although the distribution of different age groups was not substantially different between groups in the current study, it may be helpful to include age as a stratification factor in future study. Changes in gut microbiota composition occur with age [52], and it has been shown that altered gut microbiota may be associated with obesity [53], since IQP-AE-103 contains fibres and polysaccharides from okra pod and inulin, it is likely that the composition of the intestinal gut microbiota may be positively influenced by the consumption of IQP-AE-103. Hence, analysis of gut microbiota changes in overweight and obese subjects of different age groups may provide additional knowledge regarding the effect of IQP-AE-103. Moreover, appetite sensations in the study were assessed using VAS and rating scales, which were not validated for the specific use to measure changes in a similar study design. Last but not least, self-reporting of dietary intake by study subjects, applied for reasons of practicality, may be a limitation. It is known that factors such as gender and weight status affect subjects' behavior in reporting energy intake; for example, females tended to underreport compared with males, and increasing weight status was associated with an increase in underreporting of energy intake [54]. As such, results obtained in the current study, primarily evaluating weight loss, on IQP-AE-103's effect on appetite sensations remain exploratory. To evaluate a potential effect of IQP-AE-103 on food/energy intake, quantification of actual calorie intake by recording food consumption at an “ad libitum” lunch may be considered in future studies.

In the next step, the weight loss and weight maintenance efficacy of IQP-AE-103 should be evaluated in longer clinical trials. Also, it would be worthwhile to further investigate any positive effect on cardiovascular risk factors associated with obesity, particularly in subjects with elevated blood lipids. For further elucidation of mode of action, an assessment of fecal fat excretion, or postprandial blood lipid changes and potentially gut microbiome study could be considered.

## 5. Conclusion and Implication

In summary, the study results provide the first promising evidence that the intake of IQP-AE-103 causes weight loss in overweight and moderately obese subjects in conjunction with a mild hypocaloric diet. The 12-week treatment with IQP-AE-103 also showed very good tolerability. Taken together, IQP-AE-103 can be considered as a safe and effective strategy in fighting obesity with potential benefits in maintaining healthy blood lipids.

## Figures and Tables

**Table 1:** Example of a diet plan consumed daily during the trial for a 1500 kcal diet.

Calories (kcal)		Meal	Food	Portion size	Quantity (g)
400		Breakfast	Toast bread	3 slices	80
			Low-fat mozzarella (20% FDM)	1/2	62
			Fresh tomato	3 medium	150
			Olive oil	1 tsp	5
			Kiwi	1	40

100		Snack	Rye-wheat bread	1/2 slice	25
			Butter	1/2 tsp	2
			Cottage cheese (20% FDM)	2 heaped tsp	30

500		Lunch	Pork stew	1/5 portion	100
			Oil (e.g., rapeseed)	2 tsp	10
			Carrot	3 medium	150
			Onion	1 large	50
			Potato	To be weighed on a scale	350

100		Snack	Natural low fat yoghurt (1.5% FDM)	1/3 large cups	150
			Banana	1/4 medium	25
			Sesame seeds	1/2 tsp	2

400		Supper	Rye-wheat bread	2 thin slices	80
			Butter	1/2 tsp	10
			Gouda (45% FDM)	1/2 slice	15
			Turkey breast	1 slice	15
			Chicory	2 medium	150
			Orange	1 medium	100
			Honey	1 tsp	5

tsp, teaspoon; FDM, fat in dry matter.

**Table 2:** Demographic and baseline characteristics.

Parameter	FAS population		
	IQP-AE-103 LD (*N*=35)	IQP-AE-103 HD (*N*=35)	Placebo (*N*=31)
Gender			
Men	8 (22.9%)	9 (25.7%)	9 (29%)
Women	27 (77.1%)	26 (74.3%)	22 (71%)
Mean age (years)	46.8 ± 12.3	48.7 ± 12.8	46.7 ± 12.2
Age group			
18–40 years	9 (25.7%)	9 (25.7%)	11 (35.5%)
41–65 years	26 (74.3%)	26 (74.3%)	20 (64.5%)
Height (cm)	169.5 ± 10.6	168.8 ± 8.9	168.7 ± 11.0
Body weight (kg)	84.42 ± 14.89	84.04 ± 10.56	86.77 ± 15.27
BMI (kg/m^2^)	29.15 ± 2.17	29.44 ± 2.31	30.25 ± 2.59
Energy requirement (kcal)	2193.7 ± 473.9	2158.7 ± 328.0	2230.2 ± 450.6
Waist circumference (cm)	102.7 ± 10.0	100.9 ± 7.5	104.7 ± 11.4
Hip circumference (cm)	107.1 ± 7.8	106.9 ± 7.1	108.5 ± 6.9
Body fat mass (kg)	30.90 ± 6.81	30.77 ± 6.51	31.44 ± 7.34
Fat-free mass (kg)	53.52 ± 11.98	53.29 ± 9.11	55.33 ± 13.27

All data are presented as mean ± s.d. No significant difference was found between groups for all variables. FAS = full analysis set; LD = low dose; HD = high dose.

**Table 3:** Mean body weight changes throughout the study. Full analysis set.

Body weight (kg), mean ± s.d.	IQP-AE-103 LD (*N*=35)	IQP-AE-103 HD (*N*=35)	Placebo (*N*=31)
Baseline	84.42 ± 14.89	84.04 ± 10.56	86.77 ± 15.27
Week 2	83.83 ± 15.12	83.18 ± 10.35	86.43 ± 15.30
Week 4	83.18 ± 15.48	82.13 ± 10.35	85.99 ± 15.29
Week 8	82.44 ± 15.61	81.04 ± 10.35	85.89 ± 15.34
Week 12	81.41 ± 15.82	79.01 ± 9.89	85.79 ± 15.46
Baseline-week 2	0.59 ± 0.95	0.87 ± 1.16	0.34 ± 0.95
Baseline-week 4	1.24 ± 1.37	1.91 ± 1.39^a^	0.78 ± 1.44
Baseline-week 8	1.98 ± 1.81^a^	3.01 ± 1.69^a,b^	0.88 ± 1.87
Baseline-week 12	3.01 ± 2.19^a^	5.03 ± 2.50^a,b^	0.98 ± 2.06

LD = low dose; HD = high dose; ^a^significant difference vs. placebo; ^b^significant difference vs. IQP-AE-103 LD.

**Table 4:** Mean changes (reduction) in primary and secondary parameters from baseline to week 12.

Parameters	FAS population				
	IQP-AE-103 LD (*N*=35)		IQP-AE-103 HD (*N*=35)		Placebo (*N*=31)
	Mean ± s.d.	*p* values, vs. placebo	Mean ± s.d.	*p* values, vs. placebo	Mean ± s.d.
Body weight (kg)	3.01 ± 2.19	<0.001	5.03 ± 2.50	<0.001	0.98 ± 2.06
BMI (kg/m^2^)	1.09 ± 0.75	<0.001	1.75 ± 0.83	<0.001	0.34 ± 0.77
Waist circumference (cm)	2.5 ± 2.4	0.001	4.1 ± 3.3	<0.001	0.9 ± 1.6
Hip circumference (cm)	2.8 ± 2.5	0.001	4.0 ± 3.0	<0.001	0.9 ± 1.2
Body fat mass (kg)	2.62 ± 3.83	0.005	3.15 ± 2.41	<0.001	0.23 ± 2.74
Fat-free mass (kg)	0.39 ± 3.68	0.931	1.80 ± 2.38	0.124	0.75 ± 2.93

FAS = full analysis set; LD = low dose; HD = high dose.

**Table 5:** Summary of changes in laboratory measurements during the study.

Measurements	Pre- to poststudy changes			
	Low-dose IQP-AE-103 (mean ± s.d.)	High-dose IQP-AE-10 (mean ± s.d.)	Placebo (mean ± s.d.)	*p* value^*∗*^
Hemoglobin (mmol/l)	−0.08 ± 0.48	0.09 ± 0.63	−0.04 ± 0.75	NS
Hematocrit (l/l)	−0.003 ± 0.029	−0.003 ± 0.028	−0.001 ± 0.036	NS
Erythrocytes (Tpt/l)	0.01 ± 0.31	0.02 ± 0.34	−0.02 ± 0.38	NS
Thrombocytes (Gpt/l)	8.6 ± 40.5	2.3 ± 42.1	0.8 ± 56.7	NS
Leukocytes (Gpt/l)	0.08 ± 1.55	−0.33 ± 1.65	0.16 ± 1.63	NS
Mean cell volume (fl)	−0.5 ± 3.5	−1.2 ± 5.4	0.1 ± 3.8	NS
Mean corpuscular hemoglobin (fmol/Ery)	−0.021 ± 0.058	0.013 ± 0.082	0.001 ± 0.076	NS
Mean corpuscular hemoglobin concentration (mmol/l)	−0.09 ± 0.95	0.24 ± 0.87	0.03 ± 0.96	NS
Alanine aminotransferase (*μ*kat/l)	−0.075 ± 0.168	−0.035 ± 0.204	0.004 ± 0.211	NS
Aspartate aminotransferase (*μ*kat/l)	−0.172 ± 0.483	−0.137 ± 0.566	0.028 ± 0.174	NS
Alkaline phosphatase (*μ*kat/l)	0.026 ± 0.194	−0.150 ± 0.522	0.055 ± 0.305	NS
*γ*-Glutamyl transferase (*μ*kat/l)	−0.083 ± 0.205	−0.042 ± 0.161	0.052 ± 0.346	NS
Bilirubin (*μ*mol/l)	0.02 ± 6.76	4.35 ± 9.15	1.19 ± 7.89	NS
Creatinine (mmol/l)	0.26 ± 13.58	3.98 ± 9.37	1.92 ± 12.17	NS
Urea (mmol/l)	−0.17 ± 1.44	0.07 ± 1.24	−0.19 ± 1.32	NS
Uric acid (*μ*mol/l)	−1.8 ± 60.9	−6.4 ± 77.3	11.7 ± 67.2	NS
Glucose (mmol/l)	−0.01 ± 0.63	0.01 ± 0.92	−0.16 ± 0.54	NS
Vitamin A (mg/l)	−0.002 ± 0.149	0.020 ± 0.173	0.063 ± 0.142	NS
Vitamin D (ng/l)	3.51 ± 13.45	4.37 ± 10.41	3.66 ± 11.79	NS
Vitamin E (mg/l)	−0.81 ± 3.11	−0.61 ± 3.07	1.09 ± 3.78	NS
Vitamin K (*μ*g/l)	0.013 ± 1.197	0.086 ± 0.690	0.050 ± 0.689	NS
Vitamin K2mK4 (*μ*g/l)	−0.002 ± 0.152	−0.022 ± 0.154	−0.031 ± 0.114	NS
Vitamin K2mK7 (*μ*g/l)	0.143 ± 0.402	−0.157 ± 1.942	−0.086 ± 0.507	NS
Total cholesterol (mmol/l)	−0.08 ± 0.84	−0.30 ± 1.12	0.18 ± 1.09	NS
High-density lipoprotein cholesterol (mmol/l)	0.00 ± 0.39	0.10 ± 0.32	0.02 ± 0.39	NS
Low-density lipoprotein cholesterol (mmol/l)	−0.02 ± 0.77	−0.34 ± 1.04	0.03 ± 0.86	NS
Triglycerides (mmol/l)	0.00 ± 0.79	−0.09 ± 0.76	0.13 ± 0.39	NS
HbA1c (%)	0.14 ± 0.35	0.03 ± 0.34	0.11 ± 0.20	NS

^*∗*^ *p* Values from the Kruskal–Wallis test.

## Data Availability

The animal and in vitro data used to support the findings of this study are currently under embargo, whereas the research findings are commercialized. Data requests will be considered by the corresponding author 12 months after publication of this article.
