# Erratum to “Double-Blind, Randomized, Three-Armed, Placebo-Controlled, Clinical Investigation to Evaluate the Benefit and Tolerability of Two Dosages of IQP-AE-103 in Reducing Body Weight in Overweight and Moderately Obese Subjects”

**DOI:** 10.1155/2019/6189724

**Published:** 2019-07-11

**Authors:** Ralf Uebelhack, Udo Bongartz, Stephanie Seibt, Gordana Bothe, Pee Win Chong, Patricia De Costa, Natalia Wszelaki

**Affiliations:** ^1^Analyze & Realize GmbH, Weißenseer Weg 111, 10369 Berlin, Germany; ^2^Analyze & Realize GmbH, Waldseeweg 6, 13467 Berlin, Germany; ^3^Zaluvida Corporate Sdn Bhd, E-16 Plaza Mont Kiara, 2 Jalan Kiara, 50480 Kuala Lumpur, Malaysia; ^4^InQpharm Group Sdn Bhd, E-16 Plaza Mont Kiara, 2 Jalan Kiara, 50480 Kuala Lumpur, Malaysia

In the article titled “Double-Blind, Randomized, Three-Armed, Placebo-Controlled, Clinical Investigation to Evaluate the Benefit and Tolerability of Two Dosages of IQP-AE-103 in Reducing Body Weight in Overweight and Moderately Obese Subjects” [1], there was an error in the legend of Figure 1 that occurred during the production stage, as the color of the placebo results/bars has been replaced by the colors of the high dose results/bars and vice versa.

The correct version of Figure 1 should be as follows:

## Figures and Tables

**Figure 1:**
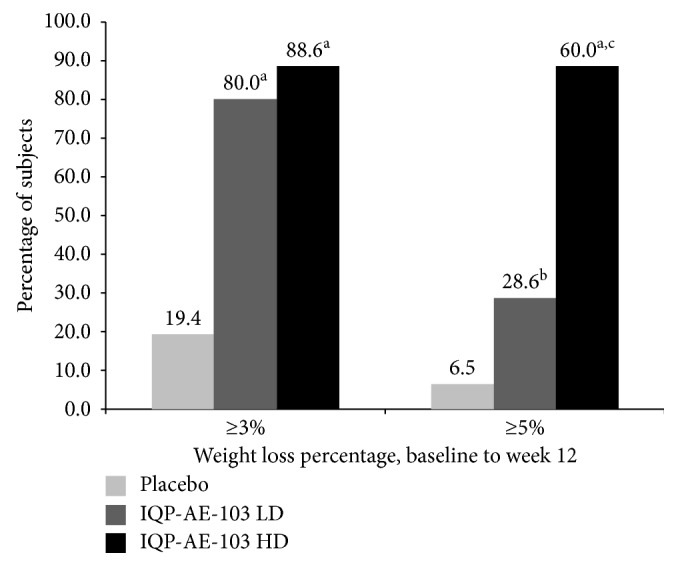
Responder rate for subjects who lost ≥3% and ≥5% of initial body weight at v6. LD = low dose; HD = high dose. ^a^*p* < 0.001 vs. placebo; ^b^*p*=0.026 vs. placebo; ^c^*p*=0.015 vs. low-dose group.
